# Predictive Values of Coagulation Parameters to Monitor COVID-19 Patients

**DOI:** 10.1155/2022/8436248

**Published:** 2022-01-31

**Authors:** Murat Seyit, Esin Avci, Atakan Yilmaz, Hande Senol, Mert Ozen, Alten Oskay

**Affiliations:** ^1^Pamukkale University, Medical Faculty, Department of Emergency Medicine, Denizli 20070, Turkey; ^2^Pamukkale University, Medical Faculty, Department of Medical Biochemistry, Denizli 20070, Turkey; ^3^Pamukkale University, Medical Faculty, Department of Biostatistics, Denizli 20070, Turkey

## Abstract

**Aims:**

In this study, we aim to unravel the relationship between coagulation parameters together with D-dimer and the severity of coronavirus disease (COVID-19) during hospitalization as well as hospitalization in the intensive care unit (ICU).

**Methods:**

This retrospective study was conducted in the Emergency Department (ED) of Pamukkale University Hospital (Denizli, Turkey) between March 1 and April 30, 2020. SARS-CoV-2 polymerase chain reaction (PCR) tests and laboratory tests, including international normalized ratio (INR), prothrombin time (PT), D-dimer, and activated thromboplastin time (APTT), were requested from 289 people presenting to the ED with symptoms of upper respiratory tract infection (URTI), such as cough, fever, and sore throat.

**Results:**

While 110 patients turned out to be polymerase chain reaction (PCR) positive, 181 individuals were PCR negative. The mean D-dimer level of the patient group was 147 ng/ml DDU (min: 9, max: 2948), and their mean PT level was found as 12.8 seconds (min: 10.3, max: 34.7). Besides, the mean APTT was 31.25 seconds (min: 19, max: 46.9), and the mean INR level was calculated as 1.09 (min: 0.88, max: 2.93). 35 of the patients were not hospitalized, while 43 were admitted to the Infectious Diseases, 20 to the Chest Diseases, and 12 to the ICU.

**Conclusions:**

It can be concluded from our findings that D-dimer, PT, and INR levels remained elevated in the COVID-19-diagnosed patients, but these parameters were unable to discriminate accurately between the patients with positive and negative SARS-CoV-2 results. Our findings also suggest that coagulation parameters might occupy a critical role in documenting clinical severity in patients with COVID-19 infection and requiring hospitalization.

## 1. Introduction

Upon the reports of unexplained cases of viral pneumonia in Wuhan, China, in the late 2019, the Chinese Center for Disease Control and Prevention (CCDC) reported a novel coronavirus through throat and nose swab samples in January [1]. Subsequently, the World Health Organization (WHO) named after this virus-borne infection as coronavirus disease (COVID-19) and declared it an urgent public health problem worldwide [2, 3]. According to the report issued by WHO on January 18, 2020, there have been 97,831,595 confirmed cases, 2,120,877 deaths, and 220 affected countries across the world [4]. By the same date, the Turkish Ministry of Health announced their data as 28,497,084 tests, 2,429,606 infected patients, and 25,073 deaths, and 1,905 severe cases [5].

Clinically, it is well-established from previous reports that thrombosis is a common complication among the cases infected with COVID-19 and among those in whom the disease show severe progression. For these cases, infection, comorbid diseases, and advanced age may be suggestive of increased risk of thrombosis. Infection-induced dysfunctions developing in endothelial cells in the disease's pathogenesis, overproduction of thrombin, blockade of fibrinolysis, and increased viscosity due to hypoxia are all included in the hypercoagulopathy of COVID-19 patients [6]. Since the diffuse intravascular coagulation occurring in COVID-19 displays a different picture from the coagulation in sepsis, this picture has started to be called “COVID-19-related coagulopathy.” Direct infection as a result of SARS-CoV-2 is documented in vascular beds of multiple organs by postmortem histological studies; in addition, apoptotic death of endothelial cells is also shown to occur in these studies [7].

Specifying some laboratory parameters while evaluating the risk of those infected with COVID-19 can help clinicians develop strategies to prevent early mortality in severe patients. Within this framework, our study aims to unravel the relationship between coagulation parameters together with D-dimer elevation and the clinical progression of the disease during hospitalization as well as hospitalization in the intensive care unit (ICU).

## 2. Materials and Methods

### 2.1. Study Design and Participants

The approval for this study was given by Non-Interventional Ethic Committee of Pamukkale University with the approval number 60116787-020/28665. This retrospective study was conducted in the Emergency Department (ED) of Pamukkale University Hospital (Denizli, Turkey) between March 1 and April 30, 2020. SARS-CoV-2 polymerase chain reaction (PCR) tests and laboratory tests, including international normalized ratio (INR), prothrombin time (PT), D-dimer, and activated thromboplastin time (APTT), were requested from 289 people presenting to the ED with symptoms of upper respiratory tract infection (URTI), such as cough, sore throat, and fever.

The medical history of the patients presenting to the ED with COVID-19 clinical suspicion was recorded, and their physical examination was performed. Their PCR samples were then collected by checking their vital parameters (respiratory rate, rhythm, oxygen saturation, blood pressure, heart rate, and body temperature). The patients with dyspnea, SaO_2_ <93% at room air, respiratory rate >28/min, PaO_2_/FiO_2_ <300, and/or > 50% increase in lung infiltration within 24 to 48 hours were followed up during their hospitalization. They were also transferred to the ICU in the case of acute respiratory distress syndrome (ARDS), severe pneumonia, acute renal failure, cardiogenic shock, arrhythmia, sepsis, septic shock, or multiorgan failure.

### 2.2. Data Collection

The demographic data, D-dimer levels, PT, APTT, and INR results of the patients were retrospectively screened from the laboratory information system, and their clinical findings were obtained from the hospital informational system. PCR test results were retrieved from the public health organization system, called “National Health Service,” organized for contagious disease monitoring.

### 2.3. Laboratory Assay and Intervention

The D-dimer, PT, and APTT levels were analyzed on ACL-TOP systems (Instrumentation Laboratory, Benford, USA) by using a latex-enhanced photometric immunoassay from the plasma samples obtained from citrate tubes. The D-dimer results were expressed in ng/mL D-dimer equivalent unit, while those of PT and APTT were expressed in seconds.

### 2.4. Statistics

All the extracted data were analyzed using SPSS v.25.0. The data distribution was checked by a Kolmogorov–Smirnov test. The continuous variables were presented as the mean ± standard deviation and median, whereas the categorical variables were provided as frequency and percent. Kruskal–Wallis variance analysis (post hoc: Mann–Whitney *U* test with Bonferroni correction) was performed for the comparison of independent groups. The ROC analysis was performed for cut-off values in order to predict SARS-CoV-2 positivity. Youden Index values were utilized to specify the optimal cut-off values. Besides, the significance level was set at <0.05.

## 3. Results

While 110 patients turned out to be PCR positive, 181 individuals were PCR negative. The median age of 110 PCR-positive patients was 42 (min: 18, max: 86), whereas 13.6% of them were over 65 years old, and 56.3% (62/110) were male.

The mean D-dimer level of the patient group was 147 ng/ml DDU (min: 9, max: 2948), and their mean PT level was found as 12.8 seconds (min: 10.3, max: 34.7). Besides, the mean APTT was 31.25 seconds (min: 19, max: 46.9), and the mean INR level was calculated as 1.09 (min: 0.88, max: 2.93).

35 of the patients were not hospitalized, while 43 were admitted to the Infectious Diseases, 20 to the Chest Diseases, and 12 to the ICU. In the meantime, Infectious Diseases and Chest Diseases are the subdisciplines of Internal Medicine.

The D-dimer, PT, and INR levels of the nonhospitalized patients as well as those admitted to other departments are presented in Table 1.

As is evident from Table 1, the D-dimer levels differed significantly between the nonhospitalized patient cohort and all the other inpatients (*P* < 0.05); however, no significant difference was evident between those hospitalized in the service and those in the ICU.

With respect to PT and INR levels, a significant difference was identified only between the nonhospitalized group and those followed up in the ICU (*P* < 0.05) (Table 1). However, PT and INR levels did not indicate any significant difference between the inpatients and the outpatients.

**Figure 1:**
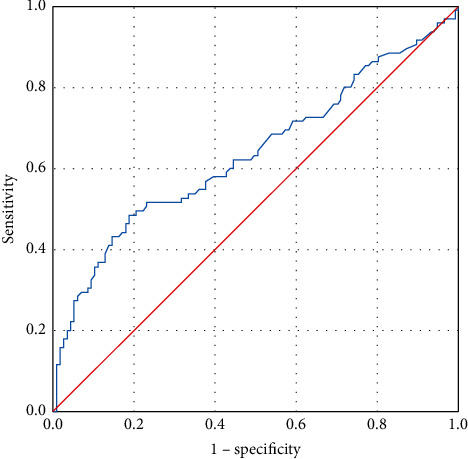
Receiver operator characteristic curve for D-dimer.

The area under the curve (AUC) for the D-dimer, PT, and INR levels was calculated as 0.637 (*P* = 0.001), 0.690 (*P* = 0.0001), and 0.704 (*P* = 0.0001), respectively (Figure 1).

All of 5 nonsurvivors in our study had been hospitalized and followed up in the ICU. The demographic and laboratory parameters of the nonsurvivors are provided in Table 2.

## 4. Discussion

Our study consisted of 110 patients who had presented to the ED with complaints of URTI and subsequently turned out to have a positive SARS-CoV-2 PCR, and their coagulopathy results were compared with the status of hospitalization in different services. The most striking result of our study is that as the D-dimer, PT, and INR levels increase, so do the hospitalization and intensive care requirements of the patients.

D-Dimer is not only an indicator of fibrin turnover and fibrinolysis but also a marker of intravascular thrombosis. D-Dimer is known as a soluble fibrin product induced by the breakdown of vascular thrombus by means of fibrinolytic mechanism. D-Dimer is most commonly used to diagnose and monitor diffuse intravascular coagulation and also to monitor thrombosis or the risk of bleeding [8]. D-Dimer produced by fibrin breakdown provides clinical evidence for the severity of the hypercoagulability condition. The body triggers coagulation mechanism to initiate physiological response in the case of an infection. It should be noted that D-dimer can also be increased in a wide range of conditions, such as infection, inflammation, cancer, previous trauma, previous surgical intervention, cerebrovascular stroke, and ischemic heart disease [9]. In our study, D-dimer levels were assessed in terms of determining the decision for hospitalization and the requirement for ICU care. Accordingly, in comparison to all the parameters we worked on, the progressive elevation of D-dimer necessitates hospitalization, and the patients are likely to require ICU care at higher D-dimer levels. When compared to the levels identified in the outpatients (mean: 91.78), the mean D-dimer scores tripled in the inpatients in Infectious Diseases (mean: 302.86), quadrupled in those in the Chest Diseases (mean: 396.85), and were more than ten-fold in the patients hospitalized in the ICU (mean: 1164.22). The most striking result revealed in our study is that, as D-dimer levels increase, so does the severity of disease and the likelihood of patients' admission to the ICU.

Since D-dimer specificity turned out to be low in our study, a specific cut-off value could not be specified, but the median value above 790 ng/ml could be a key prognostic factor in identifying the intensive care needs of these patients at an early stage (Figure 1). In addition, the PT and INR values differed significantly between the outpatients and the inpatients in the ICU (Table 1).

The optimum cut-off point, identified as the point closest to the upper left corner, was 112.5 *μ*g/mL with 54.7% sensitivity and 63.7% specificity. Area under receiver operator characteristic curve was 0.637.

In their study on survivors and nonsurvivors of 183 patients with confirmed coronavirus pneumonia, Tang et al. observed in Tongji Hospital that increased PT and APTT values, and more importantly, markedly elevated D-dimer levels were notable in coronavirus-induced deaths [10]. Likewise, our study revealed that markedly increased D-dimer levels are highly likely to require intensive care hospitalization.

In another study on 41 patients hospitalized with COVID-19 diagnosis, Huang et al. documented higher D-dimer levels in the patients requiring ICU care than in those who did not, and the median value of D-dimer level was specified as 500 ng/ml (0.5 *μ*g/ml) [11]. When it comes to our study, the D-dimer median value was calculated as 59 ng/ml among the patients with PCR positive and discharged, 170 ng/ml among the inpatients in Infectious Diseases, 222.5 ng/ml among those hospitalized in the Chest Diseases, and 790 ng/ml in those monitored in the ICU. Given the aforementioned figures, it would be safe to assume that when the D-dimer levels were elevated, the healthcare needs of the patients increased accordingly.

Conners et al. suggested that the initial coagulopathy of this disease manifested a marked increase in fibrinogen degradation products and D-dimer and that abnormalities in platelet counts, APTT, and PT developed later [12]. Likewise, the present study highlights the prominence of D-dimer elevation in the early diagnosis of COVID-19. Within this context, we observed that the patients tended not to be hospitalized if their D-dimer levels turned out to be low on admission. As these levels increased, the patients were gradually transferred to the Infectious Diseases, the Chest Diseases, and the anesthesia ICU, respectively. This leads to the plausible conclusion that the more elevated D-dimer levels are, the more severe clinical manifestations of the patient's prognosis will show.

The blood parameters of the very first 99 patients hospitalized in Wuhan were investigated by Chen et al., who documented the abnormal coagulation parameters, such as elevated levels of APTT (6% of the cases), PT (5% of the cases), D-dimer (36% of the cases), interleukin-6, and increased erythrocyte sedimentation rate, as well as c-reactive protein [13]. In our study, a marked elevation in PT and D-dimer values was observable, though the APTT levels did not reveal a significant difference. Our findings indicate that PT time differs significantly between the outpatients and the inpatients in the ICU, and we suggest that as the D-dimer levels of the patients are elevated, they should be clinically followed up more closely.

Wang et al. conducted research on 138 inpatients in Wuhan and reported that they had normal APTT and minimally elevated PT and that 26% of the ICU inpatients had higher D-dimer levels [14]. Similarly, our study documented that the APTT levels did not differ significantly among our study population, but that the only significant difference was noted in PT levels between the outpatients and the inpatients in the ICU.

Zhang et al. performed research on 343 patients with COVID-19 in the first quarter of 2020 and reported the incidence of in-hospital fatality to be significantly higher in patients with D-dimer ≥2.0 *μ*g/ml than those with D-dimer <2.0 *μ*g/ml on admission [15]. The researchers also suggested that more than a four-fold increase in D-dimer levels could be set as the threshold value that indicates mortality. However, because the specificity of the D-dimer cut-off value in our study remained low, a specific cut-off value could not be identified. Nevertheless, D-dimer elevation can be monitored closely to have an insight into the clinical severity of COVID-19 rather than its diagnosis. Furthermore, the predictive value of D-dimer can be utilized for the hospitalization of patients in the appropriate services or the ICU.

In their summary of the bulletin by the CCDC, Wu et al. stated that, out of 72,314 COVID-19 cases, there were 44,672 (62%) confirmed cases, 16,186 (22%) suspected cases, 10,567 (15%) diagnosed cases, and 889 (1%) asymptomatic cases [16]. Considering the spectrum of disease, 81% of the cases were classified as mild, 14.5% as severe, and 5% as critical (single or multiorgan failure, septic shock, and respiratory failure). The remarkable data in this study are that 2.3% of the diagnosed patients died, that the mortality rate of the nonsurvivors aged over 80 years is 14.8%, and that 49.0% of the critical cases died. In addition, the death of 1 out of 2 critical patients can be considered a serious indicator of mortality. This fatality rate is more than 3 times as much as even that of the patients over 80 years old. By contrast, 5 patients in our study, all of whom were hospitalized and followed up in the ICU, died. Moreover, D-dimer elevation was a significant factor in terms of identifying intensive care or critical patients and following them up in the ICU, and there was a significant difference between the outpatients and inpatients in the ICU with respect to INR and PT elevation. Our results can guide physicians in deciding on the clinical severity, rather than the diagnosis, of COVID-19 by considering D-dimer elevation and in establishing whether patients require ICU care or not by taking INR and PT levels into account.

### 4.1. Limitations

We are aware that the reported results in our study may have been affected by some limitations. First, our findings are based on only a limited patient population admitted to our ED. Second, the data were screened retrospectively. We hold that performing these blood tests with simpler equipment and in different healthcare centers is likely to catalyze the diagnosis of the disease. Third, it would be more appropriate to include further blood parameters to consider hospitalization and follow-up in addition to the ones assessed in this study, such as D-dimer, PT, APTT, and INR, and to reconsider them together with additional laboratory findings. Fourth, a scoring system indicating the patients' status on admission and follow-up could not be utilized. Finally, though the elevation of D-dimer in our study ran parallel to clinical severity, a clear threshold value could not be specified.

## 5. Conclusion

It can be concluded from our findings that D-dimer, PT, and INR levels remained elevated in COVID-19 patients, but these parameters were unable to discriminate accurately between the patients with positive and negative SARS-CoV-2 results. Our findings also suggest that coagulation parameters might occupy a critical role in documenting clinical severity in patients infected with COVID-19 and requiring hospitalization.

## Figures and Tables

**Table 1:** Patients' admissions to different services by their D-dimer, PT, and INR levels and between-group differences.

	Hospitalization	Mean ± standard deviation (min–max)	*P* value
D-Dimer	Nonhospitalized [1]	91.78 ± 76.55 59 (9–307)	0.0001*∗* [1, 1, 1, 2, 2, 2, 3, 3, 4]
	Infection [2]	302.86 ± 509.14 170 (18–2714)	
	Chest diseases [3]	396.85 ± 461.14 222.5 (42–1972)	
	Intensive care unit [4]	1164.22 ± 1095.05 790 (14–2948)	

PT	Nonhospitalized [1]	12.26 ± 0.8 12.4 (10.7–13.5)	0.005*∗* [1–4]
	Infection [2]	13.47 ± 3.73 12.9 (10.5–34.7)	
	Chest diseases [3]	13.29 ± 1.98 12.9 (10.3–19.1)	
	Intensive care unit [4]	14.74 ± 2.66 14 (11.9–21.3)	

INR	Nonhospitalized [1]	1.05 ± 0.07 1.06 (0.92–1.15)	0.005*∗* [1–4]
	Infection [2]	1.15 ± 0.31 1.1 (0.9–2.93)	
	Chest diseases [3]	1.13 ± 0.17 1.1 (0.88–1.62)	
	Intensive care unit [4]	1.25 ± 0.22 1.19 (1.02–1.8)	

**Table 2:** Demographic and clinical features of the nonsurvivors.

Age	Sex	D-Dimer	APTT	PTT	Hospitalization
65	Male	2948	32	15.7	Intensive care unit
83	Female	642	21.2	21.3	Intensive care unit
63	Female	2453	25.4	14	Intensive care unit
56	Female	995	29.5	12.1	Intensive care unit
77	Male	1119	30.3	14.6	Intensive care unit

## Data Availability

The data used to support the findings of this study are available from the corresponding author upon request.
